# The challenges of making informed decisions about treatment and trial participation following a cancer diagnosis: a qualitative study involving adolescents and young adults with cancer and their caregivers

**DOI:** 10.1186/s12913-019-4851-1

**Published:** 2020-01-08

**Authors:** Ruth I. Hart, David A. Cameron, Fiona J. Cowie, Jeni Harden, Nicholas B. Heaney, David Rankin, Angela B. Jesudason, Julia Lawton

**Affiliations:** 1 Usher Institute, Medical School, University of Edinburgh, Teviot Place, Edinburgh, EH8 9AG UK; 2 NHS Research Scotland Cancer Lead and Cancer Research UK Edinburgh Centre, MRC Institute of Genetics & Molecular Medicine, The University of Edinburgh, Western General Hospital, Crewe Road South, Edinburgh, EH4 2XR UK; 3 Beatson West of Scotland Cancer Centre, 1053 Great Western Road, Glasgow, G12 0YN UK; 4 Royal Hospital for Sick Children, Department of Paediatric Haematology and Oncology, Sciennes Road, Edinburgh, EH9 1LF UK

## Abstract

**Background:**

Limited attention has been paid to adolescents and young adults’ (AYA's) experiences in the aftermath of a cancer diagnosis, despite this being a time when potentially life-changing decisions are made. We explored AYA’s and caregivers’ experiences of, and views about, making treatment and trial participation decisions following a cancer diagnosis, in order to understand, and help facilitate, informed treatment decision-making in this age group.

**Methods:**

Interviews were undertaken with 18 AYA diagnosed, or re-diagnosed, with cancer when aged 16–24 years, and 15 parents/caregivers. Analysis focused on the identification and description of explanatory themes.

**Results:**

Most AYA described being extremely unwell by the time of diagnosis and, consequently, experiencing difficulties processing the news. Distress and acceleration in clinical activity following diagnosis could further impede the absorption of treatment-relevant information. After referral to a specialist cancer unit, many AYA described quickly transitioning to a calm and pragmatic mind-set, and wanting to commence treatment at the earliest opportunity. Most reported seeing information about short-term side-effects of treatment as having limited relevance to their recovery-focused outlook at that time. AYA seldom indicated wanting to make choices about front-line treatment, with most preferring to defer decisions to health professionals. Even when charged with decisions about trial participation, AYA reported welcoming a strong health professional steer. Parents/caregivers attempted to compensate for AYA’s limited engagement with treatment-relevant information. However, in seeking to ensure AYA received the best treatment, these individuals had conflicting priorities and information needs.

**Conclusion:**

Our study highlights the challenging context in which AYA are confronted with decisions about front-line treatment, and reveals how their responses make it hard to ensure their decisions are fully informed. It raises questions about the direct value, to AYA, of approaches that aim to promote decision-making by improving understanding and recall of information, though such approaches may be of value to caregivers. In seeking to improve information-giving and involvement in treatment-related decision-making at diagnosis, care should be taken not to delegitimize the preference of many AYA for a directive approach from trusted clinicians.

## Background

A growing body of research has looked at the experiences and views of adolescents and young adults (AYA) with cancer. This work has been undertaken to identify challenges and care needs that may be specific to this age group and support development of age-appropriate cancer services [1–7]. When diagnosis has been the focus of AYA research, this work has primarily been undertaken to understand (and help address) delays to diagnosing cancer (e.g. [8, 9]. Limited attention has been paid to AYA’s experiences and support needs in the immediate aftermath of diagnosis [10], despite this being the time when critical, and potentially life-changing, decisions about cancer treatments are made. Hence, gaps remain in understanding how best to support decision-making amongst AYA during the time between diagnosis and initiation of front-line treatment.

As commentators have observed, late adolescence and young adulthood is a time when significant physical, emotional and cognitive changes can occur [11]. Hence, AYA may experience different challenges following a cancer diagnosis and have different information and support needs to other age groups [7, 10, 11]. Decision-making may be affected by incomplete development of executive functioning skills, such as planning and impulse control [11]. AYA’s decision-making might also be compromised by distress, which may be heightened due to cancer being unexpected in this age group. Consequently, decisions which need to be made very soon after diagnosis, including those about frontline treatment and/or enrolment into clinical trials, may present particular challenges.

Given the varied, complex and – to most of the population – unfamiliar nature of cancer treatments [12–14], newly diagnosed individuals of all ages are often poorly equipped to make treatment-related decisions. Despite being presented with information about the practicalities, risks and benefits of proposed treatment(s), research suggests patients (in general) often struggle to understand fully important issues such as treatment intent and prognoses [12]. Where, as might be the case in a clinical trial, information encompasses two or more distinct regimens, cognitive demands may be further amplified.

AYA with cancer have lower improvements in survival rates than children and older adults and their low participation in cancer trials is believed to be a contributory factor [15–17]. This has prompted calls to identify and address barriers to trial enrolment in this age group [7, 11]. Specifically, commentators have highlighted the need to investigate AYA’s psychological response to a cancer diagnosis [18] and understand the challenges to enrolling individuals to clinical trials at this time [16, 18]. The importance of consulting caregivers and exploring their perspectives has also been noted, as these individuals may influence AYA’s decision-making [19].

Some research has now explored barriers to clinical trial participation from AYA’s perspectives. Studies have, however, been limited by questionnaire designs [20, 21], single site recruitment [22, 23] and/or because they have focused on individuals with one type of cancer [20, 24, 25]. Participant samples have also tended to be skewed towards adolescents under the legal age for independent decision-making and consent. Hence, it is unsurprising that some studies have found parents to be central drivers in decision-making [21, 22]. Due to these limitations, a recent systematic review concluded that AYA’s perceptions and attitudes towards clinical trial participation remain under-explored, especially amongst those treated in adult cancer centres [11]. In addition, it is notable that research and debates about improving trial participation by AYA have focused on how to increase enrolment, rather than the quality of their decision-making.

Here, we report findings from interviews undertaken with AYA diagnosed with cancer whilst aged 16–24 years and their caregivers. The study was conducted in Scotland, UK, where individuals aged 16+ years have the legal right to make decisions about medical procedures and treatment, including trial participation. We explored interviewees’ experiences of, and views about, making decisions about treatment and/or trial participation following a cancer diagnosis, in order to understand, and help facilitate, informed treatment-related decision-making in this age group.

## Methods

We describe our methods, below, in line with the consolidated criteria for reporting qualitative studies (COREQ) [26]. Qualitative methods are recommended when little is known about the area under investigation, as they allow findings to emerge from the data, rather than testing pre-determined hypotheses [27, 28]. We employed an inductive, semi-structured interview design entailing simultaneous data collection and analysis. This afforded participants the flexibility to raise issues they perceived as salient and allowed issues identified in early interviews, including those unforeseen at the outset, to inform the questions asked, and areas explored, in later ones.

### Theoretical framework(s)

Our work took its epistemological orientation from critical realism, a philosophy which, in simple terms, treats accounts as indicative of participants’ lived experiences and perceived realities, whilst recognising that what is disclosed in interviews is contextually-mediated and influenced by a variety of social, circumstantial and other factors [29, 30]. Our methodological orientation was qualitative description [31, 32], a pragmatic approach focused on the identification and description of minimally-theorised explanatory themes. Hence, we did not embark on data collection with allegiance to any particular theoretical concepts; however, literature on experiences of diagnosis sensitised us to the possibility that the events leading up to a (cancer) diagnosis might provide a context for, and influence and inform, individuals’ emotional reactions and subsequent treatment decision-making [33, 34].

### Context/setting

Our study was conducted in Scotland, UK. National Health Service (NHS) Scotland delivers cancer care, free of charge, through 14 health boards and more than 20 hospitals. Most patients diagnosed with cancer at age 16 or above receive treatment in an adult hospital, though paediatric hospitals deliver care to some patients aged 16–19 years. Specialist AYA units are available within the four largest health boards.

### Sampling and recruitment

Around 180 young people aged 16–24 are diagnosed with cancer in Scotland each year. From this pool of potential participants, we sought, purposively, to recruit AYA with varying characteristics in terms of age, gender, diagnosis, place of care, and trial experience. Clinical teams facilitated the research by identifying AYA diagnosed, or re-diagnosed, with cancer when aged 16–24 years from three paediatric and five adult cancer centres in Scotland. Initial contact with AYA patients was made by members of their direct care team, who offered these AYA recruitment packs containing an opt-in form returnable to the qualitative research team. Due to the range of direct care colleagues involved in this process we cannot say definitively how many AYA were approached, or how many chose not to take part.

Where opt-in forms were returned, contact was made by the project researcher (RIH) who talked through the details and practicalities of taking part in the research. AYA who agreed to take part in an interview were asked to give a recruitment pack to a caregiver (e.g., a parent or partner) or another individual (e.g., a friend) who had been influential in their decision-making about cancer treatment and/or care. Again, these packs included opt-in forms, on receipt of which contact was made by RIH. All AYA were recruited between November 2017 and December 2018, whilst caregivers were recruited between February 2018 and January 2019.

### Data collection

Interviews were undertaken by RIH, an experienced (non-clinical) qualitative researcher, at a time and location convenient to participants – typically their own homes or the hospital at which AYA were receiving care. In both such locations, interruptions by family members and/or healthcare professionals were relatively common. In such situations, interviews were generally paused, and resumed once privacy was restored. While AYA and caregivers were invited to take part in separate interviews, four AYA elected to be interviewed with one or more parent-caregiver. Topic guides helped ensure the discussion remained relevant to the study aims, while affording scope and flexibility for participants to raise issues they perceived as salient, including those unforeseen at the outset. Key areas explored relevant to the reporting in this article are outlined in an Additional file 1: (a Microsoft Word document, with the file extension .docx). In developing topic guides we drew on the expertise of clinical co-investigators and AYA advisors, as well as relevant literature. Topic guides were revised in light of emerging findings and adapted in situ, i.e. used flexibly, to take account of variations in age, development, education and interviewees’ emotional states, and to probe and explore in more depthissues which particular participants chose to disclose. Whilst our goal was to elicit rich information, we were cognisant of the responsibility not to cause avoidable/unnecessary distress. Hence decisions were made in some instances not to pursue certain issues in depth. Interviews typically lasted 1–2 h; all were digitally-recorded and transcribed, with interviewees’ consent. Information on the context and non-verbal components of the interview were recorded in field notes. Data collection continued until our sampling ambitions had been broadly satisfied and no new findings were identified in new data collected (data saturation).

### Data analysis

Two highly experienced, non-clinical qualitative researchers (RIH and JL) analysed the data, using the method of constant comparison [35] to identify key themes in AYA and caregiver accounts. Both researchers immersed themselves in the data and read interviews through repeatedly, before independently undertaking preliminary analyses. They wrote separate reports and then met to discuss their interpretations and agree on a coding frame which captured key themes. Coded datasets were subjected to further analyses to allow more nuanced interpretations of the data and identification of illustrative quotations, with a qualitative data-indexing package, NVivo (Version 11, QSR International Pty Ltd., Doncaster, Victoria, Australia), used to facilitate data coding and retrieval. Emerging findings, supported by illustrative quotations, were shared with the wider research team, members of the study advisory groups, and participating AYA and their peers (via a workshop convened in the final phase of the project). Feedback was largely confirmatory and was used to inform study recommendations.

## Results

18 AYA were interviewed, of these 13 nominated one or more caregiver who was also interviewed (11 mothers, 3 fathers and 1 partner). The remainder either did not wish to involve a caregiver or felt unable to do so (e.g. as their family resided in another country and/or spoke limited English). See Table 1 for more details about the sample. As participants were drawn from a very small population and are, therefore, potentially easy to identify, we have only been able to provide limited clinical and personal information in our reporting, below.

**Table 1:** Characteristics of study participants

AYA with cancer (*n* = 18)		
Age: median (range)	At diagnosis	19 (16–24) years
	At interview	20 (17–26) years
Gender, male: n (%)		14 (78%)
Ethnicity: n (%)	White British	14 (78%)
	Non-white British	2 (11%)
	White non-British	2 (11%)
Education/employment at diagnosis: n (%)	Employment / work-based training	6 (33%)
	School / college	6 (33%)
	Undergraduate studies	3 (17%)
	Not in education / employment	3 (17%)
Diagnosis: n (%)^a^	Bone sarcoma	6 (33%)
	Leukaemia	4 (22%)
	Germ cell tumour	3 (17%)
	Other sarcoma	2 (11%)
	Lymphoma	1 (6%)
	CNS tumour	1 (6%)
	Melanoma	1 (6%)
Diagnostic type: n (%)	Primary cancer	16 (89%)
	Relapsed cancer	2 (11%)
Place of care: n (%)^a^	Adult hospital with AYA unit	14 (78%)
	Paediatric hospital with AYA unit	3 (17%)
	Adult hospital without AYA unit	1 (6%)
Reported enrolment in a trial: n (%)		5 (28%)
Interviewed independently of caregiver(s): n (%)		14 (78%)
Caregivers (*n* = 15)		
Relationship to AYA: n (%)	Mother	11 (73%)
	Father	3 (20%)
	Partner	1 (7%)
Ethnicity: n (%)	White British	14 (93%)
	Non-white British	1 (7%)
Own occupation at AYA’s diagnosis: n (%)	Professional	8 (53%)
	Semi-professional / Skilled	6 (40%)
	Unskilled	1 (7%)

^a^Percentages do not sum to 100% due to rounding

Below, we begin by describing AYA’s reactions to diagnosis and their experiences in its immediate aftermath, in order to illuminate the context in which they were confronted with decisions about their treatment. Building on this, we document AYA’s responses to treatment-relevant information at this time, and identify reasons why these individuals did not choose, or push, to be actively involved in decision-making. We then consider how caregivers sought to play support(ive) roles to help compensate for AYA’s lack of engagement with information and ensure AYA’s clinical interests were met. Key themes which structure our reporting include: difficulties processing the news; a rush of emotion; a whirlwind of activity; struggling to absorb information; going into a (recovery-focused) zone; disengaging from challenging information; detaching from decision-making; seeking to protect and support AYA; and conflicting information needs.

### Difficulties processing the news

AYA and caregiver participants described a variety of circumstances and events which had led to a cancer diagnosis and referral to a specialist cancer unit. Most reported having been made aware that the results of key tests (such as MRI scans and biopsies) indicated that they/AYA might or did have cancer by a health professional other than an oncologist (e.g. a surgeon). Many described themselves/AYA as having experienced marked deteriorations in health by this point. Some AYA commented that they had felt so unwell, overwhelmed, and exhausted that they had struggled initially to process the news. A16, for example, a young man eventually diagnosed with osteosarcoma, described having been in “unbearable pain” when cancer was first broached, and how, as a consequence, “I didn’t process the information… I wasn’t absorbing anything.” Others described how the after-effects of a general anaesthetic and/or initiation of pain relief had interfered with their ability to process what they were being told. This included A14, a young man diagnosed with a form of sarcoma uncommon in young adults. A14 reported how, when first advised that his symptoms were suggestive of this rare cancer, he had been “so dosed up on morphine that I had no idea what that [diagnosis] meant.”

### A rush of emotion

In the majority of cases, shock and extreme distress were described as AYA’s over-riding reactions to the news that they might, or did, have cancer. As the mother of a young man diagnosed with a haematological malignancy recalled: “the first thing (A09) said to me, he said, am I going to die? And I just remember tears pouring down his face, so he did get a big fright.” Notably, however, some AYA participants also described having felt relief, due to the severity of their symptoms and the prospect of now receiving the correct treatment. This included A05, a young woman ultimately diagnosed with bone cancer, who reported presenting to her GP and other professionals on repeated occasions before finally being diagnosed. This young woman described having eventually gone to an accident and emergency department (A&E) in a state of desperation and extreme pain: “I was just in agony. I didn’t sleep, couldn’t eat”. Hence when a series of tests revealed she had a tumour, she described experiencing a range of conflicting emotions:

“I’m not going to lie… I was a wee bit stunned and then I was really upset, we were all crying and then I was a bit like, what’s going to happen… but then part of me, in a weird way was like, this is gonna take the pain away. Like the pain was that bad.”

### A whirlwind of activity

In many cases, the sudden acceleration in activity which followed diagnosis left little time for reflection, and presented additional challenges to AYA processing the news. Many participants reported how they/AYA were sent straight onto a specialist cancer unit, sometimes within a matter of hours of receiving their initial results. As A05’s father recounted in a joint interview with his daughter:

“things just moved so quickly... it was like a whirlwind. It just seemed to go from him telling us, and us trying to take it in, and then we had to get to (Children’s Hospital) to meet with (consultant oncologist) and then things just went very, very quickly.”

Participants also reported a further escalation of activity following arrival at the cancer unit, wherein, after an initial consultation, most underwent a battery of tests and medical procedures in quick succession to confirm (the type of) cancer and/or establish its spread before treatment could be determined and initiated:

“after I saw my (Consultant Oncologist) for the first time, he arranged loads of different scans Because it was very common for it to, have spread elsewhere in the body, particularly the lungs, and possibly the bones. Obviously that was extremely harrowing. I got a CT scan, I got a bone scan, I got a full body scan, heart scan, kidney scan.” (A04)

### Struggling to absorb information

AYA participants were generally able to recall some elements of the consultation in the cancer unit where test results and treatment plans were first discussed. However, many noted how, due to extreme exhaustion, the shock of diagnosis, and the limited time they had had to process the news, they had struggled to absorb and engage with treatment-relevant information. A18, for instance, described a meeting with her oncologist shortly after surgery to remove a large pelvic mass: “We were obviously talking about treatment (radiotherapy). But I’d just come out from surgery, so I was too tired”. This situation was confirmed by her mother who noted how:

“she was exhausted and I think a lot of the time (daughter) was leaning on me zonked out … she didn’t really, really understand the full implications of what it [treatment] was going to entail.”

### Going into a (recovery-focused) zone

Notwithstanding their harrowing experiences around the time of diagnosis, most AYA participants described transitioning, relatively quickly, from a mind-set of shock, extreme distress and fear, to one which had been calm, largely devoid of emotion, and deeply pragmatic. This included A12, a young man diagnosed with acute lymphoblastic leukaemia, who noted how, when a consultant discussed his diagnosis and its implications:

“Mum and dad started crying... but I didn’t, I just sat on my bed and was quite matter of fact about it… I’d had a little cry earlier, when I’d seen the (Teenage Cancer Ward) sign or whatever, but I’d kind of accepted my fate, already.”

A03, a young woman with Ewing’s sarcoma, likewise described having entered a “zone” following her arrival at the cancer unit, wherein her focus had rapidly shifted from shock and distress to recovery, and commencing treatment at the earliest opportunity to achieve this:

“you kind of just go into like a zone, you’re like, it is what it is … you know, you need to get on with it, either way, so. he sat and he explained everything [test results] to me, and I was just like right, I just wanted to get on with it, just let’s go, I wasn’t like upset or anything like that… probably when I’m finished I will be like, oh my God, how the hell did I do that? But when you’re in that zone, you’re like right, this is it, I just need to focus on getting better.”

Some participants noted how this mind-set had been fostered and enabled by the speed with which investigations had been undertaken and results had come through. Indeed, several individuals described having actively welcomed this forward momentum and accelerated activity, precisely because it had left little time to worry about their prognosis and fixate on negative scenarios:

“I’ve been lucky in the sense that. it’s just been literally full steam ahead … and I’ve preferred it like that because you’ve not got any time to think about things because it’s just like, right you’ve been diagnosed, we’re starting treatment we can start next week … it doesn’t give you time to think, like ‘oh no, this is what I’ve got, like, what’s going to happen to me?’ You know, all that stuff that’s negative.” (A16)

Positive steers from health professionals were also described as having been welcomed and as having had a galvanising effect:

“It was all really positive stuff, it was never anything really negative at all actually which suited me… From day one, he said to me, going to get you better, going to beat this kind of thing… So, I just wanted to get started then and there.” (A16)

### Disengaging from challenging information

In light of their focus on recovery and attendant wish to get on with treatment, most participants reported seeing information about short-term side-effects of treatment (e.g. hair loss, nausea) as having had limited relevance to their thinking and priorities at the time. A14, for instance, a young man diagnosed between leaving school and starting university, reported how he had not “really given a damn about short-term side-effects, the things that do matter to me are the long-term side-effects, what’s my life going to look like in five years, 10 years.”

Some AYA participants also described having chosen to disengage from potentially distressing information, despite its potential relevance to treatment decision-making, due to its potentially detrimental emotional impact. For example, A18, the young woman whose early experiences were described above, remarked: “like chemo and that… I was just too sad to read them [leaflets]… Yeah just, I’d just end up crying”. This response appeared especially marked in relation to information about prognosis. Some AYA described themselves as having had no desire for detailed information on their prospects, and appeared keen to prevent or circumvent negative thinking, as A16 explained:

“he (consultant oncologist) never told me what stage of cancer I was in, I never asked, he never mentioned once survival rates, anything like that, which I was happy with.”

### Detaching from decision-making

Though many decisions had to be made about AYA’s treatment, the majority of AYA participants saw themselves as having had limited opportunities for involvement. As one young man described, “decision-wise… it was all laid out for me in a way, there wasn’t really a lot of room for negotiations” (A15). A similar experience was recounted by A14:

“I wasn’t presented with much of a decision when I saw (oncologist)… He explained that he wanted to treat me under a regimen which was developed as part of a clinical trial… He basically told me that you’ll have a longer duration of treatment, but it substantially increases your chances of a cure and I was like, fine.”

None of these individuals questioned or challenged this directive approach in retrospect. To the contrary, AYA participants often described having preferred to defer decision-making to health professionals, due to having felt unwell and overwhelmed, and just wanting to get on with treatment. A particularly poignant example was provided by A17, a young man diagnosed with a brain tumour following an emergency admission after collapsing at home. Having undergone surgery to remove and biopsy the tumour, this individual described his state of exhaustion as extreme: “I was sleeping after it for about 20, 22 hours per day.” He also described having felt “gutted” on discovering how advanced his brain tumour was (stage IV). A17 made it clear that, at the time, he had just wanted to be looked after and for trusted specialist health professionals to make decisions for him: “do what’s best for me, that’s what I would say.”

#### Enrolling without fully understanding

When AYA participants had been invited to take part in a clinical trial following diagnosis, more concrete and tangible decisions had needed to be made. As those participants (*n* = 5) who recalled such an approach as having taken place reported, discussions about treatment and trial participation had taken place concurrently. This was because decisions about trial participation had needed to be made rapidly, before treatment could commence. In keeping with the above accounts, these individuals described how trial-participation decision-making had taken little account of information and had involved limited deliberation. The following interviewee, for example, one of the youngest in our sample, reflected on how:

“It was more like a gut [reaction], I mean, I did ask (Mum) and (Dad) what they were thinking, but I didn’t really care, it was more just, it’s probably the right thing to do. So, I just did it.” (A07)

Indeed, rather than engaging with trial-related information, participants described taking cognitive short-cuts and basing their decision on the recommendation of, or a strong steer from, the consultant who recruited them:

“I didn’t really think about it properly, because… the main factor was actually that, I trusted Dr (Name), and, he seemed keen on it, and he seemed to be wanting to persuade me to do it, so, I was quite happy to take part, without maybe knowing fully, what it entailed.” (A12)

Some noted how, as a consequence, their understanding of the trials into which they had been recruited had been very limited, as A01 explained:

“it was all a bit of a blur at that point … I didn’t quite have an understanding of the trial. To be honest I don’t quite have an understanding of it now.”

None of these individuals expressed regret about trial enrolment, and all described feeling that their health professionals had acted in their best clinical interests. However, some AYA did question in hindsight whether they had made fully informed decisions about taking part. As these individuals suggested, the problem had not resulted from the information they had been given at trial enrolment but, rather, the context and timing of the approach:

“Maybe, the bombarding of information in the first week. It’s a lot of information to take in, and then to start treatment so quickly…. Erm… so yeah like, to spread it out over a longer period of time, so that people have that time to process all of it.” (A01)

### Caregivers: seeking to protect and support AYA

Caregivers, who were mostly parents, described having wanted to do everything they could to protect and support AYA patients following a cancer diagnosis. Many reported concerns that – as AYA themselves had suggested – their son/daughter had been unable or unwilling to engage with information and actively participate in consultations at the time treatment plans were discussed. This included the mother of one young man (A10), who had been in employment and preparing to buy his first house at the time of diagnosis. M10 observed how her son, despite leading a relatively independent and adult life:

“wasn’t well, he wasn’t taking part in the conversation, it was probably more me. And I think he was just exhausted. And obviously a bit, well, scared.”

Likewise, A12’s mother noted how her son had been, “quite unwell really, (so) even like concentrating on reading documents (was) hard for him.” Reflecting further, she added: “at times I felt that (son) didn’t have the questions to ask, because (son) wasn’t well enough to be asking questions.”

Other parents described how, because of their worries that AYA had been unable to concentrate and had not wanted to deliberate at length over decisions, they had found it very challenging that it had been the young person, rather than themselves, to whom treatment-related information had been cascaded, and, moreover, who had been charged with responsibility for decision-making. As the mother of A07, the 16-year-old whose “gut” decision to participate in a trial was described earlier, explained:

“ultimately the decision, on the trial, was (son's). I struggled with that… I struggled with the fact that (Consultant) initially was talking to (son), because he can give consent, he doesn’t need our input at all. But I didn’t feel (son), after being told that overwhelming news, was kind of, you know, he needed support to help him make the decisions.” (M07)

#### Acting as retainers, investigators, sounding-boards and influencers

In response, parents/caregivers described having undertaken various overlapping support roles to help ensure AYA’s clinical interests were met. Specifically, they described having made efforts to be present in consultations to assimilate information on AYA’s behalf; for instance, by listening carefully and taking detailed notes. These individuals also described how, following consultations, they had gone away and carefully read all the written materials which AYA were given, in order to come back and ask targeted and focused questions on their behalf.

Most parents/caregivers also reported having undertaken their own investigations before any treatment or trial participation decisions were finalised. This included researching the oncologist’s credentials, finding out more about cancer and potential treatments, and/or researching trials online (when these were offered to AYA). In some cases, parents/caregivers had sought advice from personal contacts who had specialist cancer knowledge. Parents/caregivers described having undertaken these investigatory roles to help ensure AYA received the best care from the most qualified individuals, and to lobby for changes if necessary:

“Because we had a few days before we met the oncologist, I was able sort of to have my own questions as to what they were gonna do, and how they were gonna do it, and were they the best?” (M06)

Caregivers also noted how, by virtue of having undertaken their own research, they had been better placed to act as sounding boards before decisions about treatment/trial participation were finalised and consent forms signed. To this end, they also noted how they had been well situated to nudge, sway and/or endorse decisions as necessary. A01’s mother for instance, described having encouraged and supported her daughter’s decision to take part in a trial after carefully reading all of the documentation and being reassured that her treatment and care would not be adversely affected:

“when you sat down and got the information, it was only the method of treatment that was differing, it didn’t affect the outcome and that was the most important thing to me… so… I said, well you know, it’s up to you at the end of the day, it’s your body, I said, but I think it’s a good idea. So [my role in the decision-making process] was supportive.” (M01)

While these caregivers generally endorsed (trial participation) decision-making, they also noted that if they had had any concerns, they would have attempted to nudge AYA into making a different decision. As A07’s father described:

“We did have a friend who’s an oncologist… She’d gone away and done her own research, came back and said, It is a good trial… it’s probably a good one to go on – if she’d come back and said something different, we might have tried to talk (son) out of it.” (D07)

### Conflicting information needs

In undertaking these support(ive) roles, parents/caregivers recognised that there were potential conflicts between their own information needs and those of AYA. While AYA wanted to maintain a positive, recovery-focused outlook, parents/caregivers described needing realistic information, including information about AYA’s prognosis, to help ensure the best decisions were made:

“it might be that some doctors are more upfront about it, but Dr (Name)... was wholly positive with his demeanour, and I actually asked him, ‘cause I didn’t want to ask him in front of (son), I asked him, I pulled him outside that first day, and I said, with (son's) permission I asked him, You know, is, could he die from this?” (M12)

As parents further noted, this could sometimes mean treading a delicate line between satiating their own needs and requesting information which could cause distress to their child. A particularly poignant example was provided by M06, whose son had been diagnosed with an extremely rare form of cancer. M06 had been very anxious to ensure her son received his care from health professionals with prior expertise of treating his kind of cancer, even if this meant moving to another cancer unit. To do this, she had found it necessary to ask very difficult questions (e.g. about survival rates) which, as she realised, could cause her son upset:

“Because they did say they haven’t had anybody like him, which was a bit alarming, because I felt, well, how do they know how to treat him… I asked, well why isn’t he going to (City) where obviously they have got more experience with that type of tumour… I asked was there any place in Scotland where there’d been more patients that had had it? And what was the outcome? But it’s hard saying that when he’s sitting there… You don’t want to say in front of them: What’s the success rate? And you don’t want to put any more pressure on (son) by asking too many things.”

## Discussion

This study has highlighted the profoundly challenging context in which AYA diagnosed with cancer find themselves confronted with decisions about front-line treatment and/or trial participation. In keeping with other studies [21, 22, 24], we have shown how the physical effects of cancer and the shock and distress of diagnosis can influence AYA’s initial response to diagnosis, and hamper their ability to process difficult news at this time. In addition, our study has highlighted how intense emotion, and the escalation of clinical activity that follows diagnosis, can further impede AYA’s ability to absorb and process important (treatment-relevant) information. Notably, we have drawn attention to how AYA may quickly transition from a state of shock and distress to a mind-set focused on survival and recovery, which our participants described as “going into a zone”. Whilst in the zone, AYA described wanting and valuing opportunities to filter out negative scenarios. Although this mind-set may act as an important coping strategy in the aftermath of a cancer diagnosis, we have shown how it can further compromise engagement with treatment-relevant information, including information about prognosis and treatment side-effects, and thereby undermine informed decision-making. In general, we found AYA’s interest in engaging in decision-making about front-line treatment to be low, with most indicating a clear preference for a strong professional steer at this time. Caregivers expressed concerns about the quality of AYA’s decision-making, and described attempting to compensate for their limited engagement with relevant information. However, in seeking to support AYA, and help ensure that they received the best treatment/care, these individuals could have conflicting priorities and information needs.

As our findings suggest, professionals tasked with facilitating AYA’s involvement in decision-making about (front-line) treatment and/or trial participation are often confronted by major challenges in the form of AYA’s physical and emotional states at and in the immediate aftermath of a cancer diagnosis. Such challenges have led commentators to question whether it is really possible to obtain informed decisions about, and consent to, front-line treatment and trials at this time. With regard to trials, some have even asked whether it is (ethically) appropriate to attempt to recruit patients so soon after diagnosis, and where decisions about participation need to be made rapidly, so that treatment might commence [16, 22–24]. This issue of how to achieve informed decisions and/or consent under pressure is of considerable wider interest, with challenges highlighted in a range of studies. This includes work involving parents of younger children diagnosed with cancer, where, again, treatment often needs to be initiated very soon after diagnosis [36]. Approached about trial participation within hours or days of learning their child had cancer, these parents similarly struggled to assimilate and reflect upon (complex) information needed to make informed decisions. As with our AYA participants, this was due to upset, shock, and the limited timeframes available [36]. Similar concerns have also been reported in studies exploring the issues arising when recruiting individuals into (non-cancer) clinical trials in other acute/emergency situations, where again distress may be heightened and decisions need to be made quickly [37–39].

The literature offers some pointers as to potential ways of overcoming the challenges presented by poor health, distress, and short timeframes. However, while some of these proposals have been recommended for use in AYA and/or paediatric populations newly diagnosed with cancer, they are, as yet, of unproven efficacy. These proposals include using novel communication strategies (e.g. audio or video platforms), decision-aids and question prompt lists [11, 17, 40–43]. It has also been suggested that information could be given out in smaller quantities, over varied periods, and that investigators should systematically ask individuals to recall the information given when decisions are confirmed, and consent taken [36]. It is noteworthy that all such approaches focus on improving understanding and recall of information (e.g. about the risks and benefits of treatment and/or trial participation). Given our own findings that AYA may be too unwell to assimilate and recall information needed to make fully informed decisions, and may be unwilling to engage with negatative scenarios (e.g. about prognosis and treatment side-effects), these approaches may be of limited value for AYA patients confronted with decisions at diagnosis. Moreover, because of the expediency with which their cancer treatment often needs to commence, approaches that require information to be delivered at several time points may be less feasible than for other patient groups presenting with less acute forms of disease.

AYA participating in our study described how, following diagnosis, there had seldom appeared to have been any major treatment-related decisions for them to make. Agreeing to (or declining) the course of action proposed by professionals was not typically viewed as a real choice/decision. This finding is perhaps unsurprising, given that observational work undertaken in cancer multi-disciplinary team meetings has demonstrated that health professionals tend to reach a consensus about which treatment is best before delivering this recommendation to the patient [44]. Other research has indicated that, in oncology, it is common practice for clinicians to make explicit recommendations, and for there to be less scope for negotiation of treatment plans than in other clinical specialisms [45]. Hence, shared decision-making in oncology, especially in situations where professionals believe there is a course of action which is in a patient’s best clinical interests, has been reported to be rare [46]. Notably, none of the AYA in our study questioned, in hindsight, the validity and acceptability of the directive approach they described; to the contrary, these individuals gave little indication of wanting treatment choice at this time. Whilst, as others have suggested, this preference might be due to AYA having not yet fully developed executive functioning skills [1], our findings suggest more complex multi-factorial explanations, in which disease acuity and the limited time available to make decisions play important roles. Indeed, it is relevant to note that amongst other patient groups where quick treatment decisions have also needed to be made, similar preferences have been highlighted to those reported by AYA. For example, in a study involving adults with haematological malignancies who were in life-threatening situations and under extreme emotional strain, it was found that these individuals similarly leaned towards directive approaches [47]. Some commentators have argued for greater recognition of the legitimacy of this preference for a directive rather than a shared decision-making approach [48]. Mol [49] problematizes the common framing of choice as the ideal, positing that choice does not necessarily result in good care, and may leave patients feeling burdened and/or with an unhelpful illusion of control. Even key proponents of shared decision-making have acknowledged that such an approach may not be appropriate or feasible in all decisional contexts, and in some a more directive (paternalistic) approach may be both preferred and required [50, 51].

While a directive approach may be acceptable when health professionals are clear about the most efficacious and acceptable form of treatment, it is potentially more controversial in the context of clinical trial recruitment, where, in principle, equipoise exists and there is uncertainty with regard to which type of treatment is in an individual’s best clinical interests. In these situations, it may be problematic to involve consultants in trial recruitment, given that AYA tend to base their decisions upon trust in those individuals, as opposed to on careful engagement with trial-relevant information. It may be even more problematic still to follow the recommendation, made by others, that those staff to spend as much time as possible with AYA to develop rapport, in order to improve trial recruitment [16, 24]. Rather, consideration could be given to using more neutral parties in the information-giving and consent process for trials [36]. Input from psychological services might also be considered to help reduce distress following a cancer diagnosis, and, through this, potentially to increase decisional involvement by AYA [40].

The role of caregivers in such situations is both interesting and potentially important. Our study extends understanding of parent-caregivers’ concerns, and the work they undertake to support AYA. Previous work involving parents of younger children has shown that these individuals often adopt advocacy and investigator roles to help ensure the best decisions are made for their child [19, 52, 53]. Our study demonstrates that, once “children” reach the legal age to make their own decisions about treatment, parents often continue to play supportive roles. These include: attending consultations; asking questions on AYA’s behalf; undertaking research; acting as sounding boards; and, “nudging” AYA towards (different) decisions. Future research could explore the best ways of engaging this support, while being sensitive to the fact that parents’ priorities and information needs may conflict with those of AYA. Any such work must further recognize that, through choice or circumstance, not all AYA have parental support following a cancer diagnosis.

### Strengths and limitations

Using a flexible and open-ended approach, we have been able to bring new and important insights to the literature. The inclusion of a caregiver alongside an AYA perspective has revealed a more complex decision-making dynamic than erstwhile recognized. In the context of joint interviews, AYA and caregivers operated as co-producers of knowledge, with caregivers helping AYA to fill gaps in recall arising from acute illness and distress. However, in some instances, this might have inhibited open discussion about the difficulties and worries experienced (by AYA or caregivers). While we attempted to achieve heterogeneity in our AYA sample, we interviewed more males than females. Some of the stoical reactions reported may therefore reflect cultural and gendered norms and expectations regarding masculinity. Because participants’ accounts were retrospective, they may have been subject to recall bias. Hence, future prospective (longitudinal) research could be considered, including observation of consultations where key discussions about treatment/trial participation take place.

## Conclusions

Treatment in the field of AYA cancer care is guided by complex and evolving protocols, often covering long periods of time. Hence, even under optimal conditions, the cognitive demands of absorbing, processing and employing treatment information for the purposes of decision-making are substantial. Our findings indicate that, due to the context in which AYA are confronted with decisions about front-line treatment, including, where available, treatment through a trial, their decision-making may not be fully informed. The survival/recovery-focused mind-set AYA may adopt as a coping strategy further runs counter to meaningful engagement with information and decision-making. Hence when AYA are first diagnosed with cancer, rather than delving into the detail of the treatment and/or trial, they may prefer to make decisions directed by a trusted clinician. Though this preference may be viewed/interpreted differently on account of their age, there is ample evidence to suggest that AYA are far from unique in favouring a directive approach. Care should be taken not to delegitimize this preference/choice in the process of developing and implementing strategies to improve information-giving and encourage meaningful involvement in decision-making.

## Supplementary information

**Additional file 1.** Key areas explored in the AYA and caregiver interviews.

## Data Availability

The datasets (generated and/or reported in this paper are not publicly available due to the very small number of AYA diagnosed with cancer in Scotland each year. This means that it may be possible even after the removal of obvious identifiers (e.g. names and locations) for some individuals, such as health professionals providing relevant services, to identify the young people who took part. These datasets are available from the corresponding author on reasonable request.

## Abbreviations

A&E: Accident and Emergency Department
AYA: Adolescent and Young Adult
NHS: National Health Service
UK: United Kingdom

## References

[1] Patterson P, Millar B, Desille N, McDonald F (2012). The unmet needs of emerging adults with a cancer diagnosis: a qualitative study. Cancer Nurs.

[2] Lie NE, Larsen TM, Hauken MA (2018). Coping with changes and uncertainty: a qualitative study of young adult cancer patients’ challenges and coping strategies during treatment. Eur J Cancer Care.

[3] Gibson F, Hibbins S, Grew T, Morgan S, Pearce S, Stark D (2016). How young people describe the impact of living with and beyond a cancer diagnosis: feasibility of using social media as a research method. Psycho-Oncol..

[4] Grinyer A (2009). Contrasting parental perspectives with those of teenagers and young adults with cancer: comparing the findings from two qualitative studies. Eur J Oncol Nurs.

[5] Kyngäs H, Mikkonen R, Nousiainen EM, Rytilahti M, Seppänen P, Vaattovaara R (2001). Coping with the onset of cancer: coping strategies and resources of young people with cancer. Eur J Cancer Care.

[6] Foster RH, Brouwer AM, Dillon R, Bitsko MJ, Godder K, Stern M (2017). “Cancer was a speed bump in my path to enlightenment:” a qualitative analysis of situational coping experiences among young adult survivors of childhood cancer. J Psychosoc Oncol.

[7] Tai E, Buchanan N, Eliman D, Westervelt L, Beaupin L, Lawvere S (2014). Understanding and addressing the lack of clinical trial enrollment among adolescents with cancer. Pediatrics..

[8] Miedema BB, Easley J, Hamilton R (2006). Young adults' experiences with cancer: comments from patients and survivors. Can Fam Physician.

[9] Gibson F, Pearce S, Eden T, Glaser A, Hooker L, Whelan J, Kelly D (2013). Young people describe their prediagnosis cancer experience. Psycho-Oncology..

[10] Bibby H, White V, Thompson K, Anazodo A (2017). What are the unmet needs and care experiences of adolescents and young adults with cancer? A systematic review. J Adolesc Young Adult Oncol.

[11] Forcina V, Vakeesan B, Paulo C, Mitchell L, Bell JA, Tam S, Wang K (2018). Perceptions and attitudes toward clinical trials in adolescent and young adults with cancer: a systematic review. Adolesc Health Med Ther.

[12] Yousuf Zafar S, Alexander SC, Weinfurt KP, Schulman KA, Abernethy AP (2009). Decision making and quality of life in the treatment of cancer: a review. Support Care Cancer.

[13] Katz SJ, Belkora J, Elwyn G (2014). Shared decision making for treatment of cancer: challenges and opportunities. J Oncol Pract.

[14] Chen S (2017). Information behaviour and decision-making in patients during their cancer journey. Electron Libr.

[15] Bleyer A, Montello M, Budd T, Saxman S (2005). National survival trends of young adults with sarcoma: lack of progress is associated with lack of clinical trial participation. Cancer..

[16] Fern L, Davies S, Eden T, Feltbower R, Grant R, Hawkins M (2008). Rates of inclusion of teenagers and young adults in England into National Cancer Research Network clinical trials: report from the National Cancer Research Institute (NCRI) teenage and young adult clinical studies development group. Brit J Cancer.

[17] Friend BD, Baweja A, Schiller G, Bergman J, Litwin MS, Goldman JW (2017). Clinical trial enrollment of adolescent and young adult patients with cancer: a systematic review of the literature and proposed solutions. Clin Oncol Adolesc Young Adults.

[18] Freyer DR, Seibel NL (2015). The clinical trials gap for adolescents and young adults with cancer: recent progress and conceptual framework for continued research. Curr Pediatr Rep.

[19] Snethen JA, Broome ME, Knafl K, Deatrick JA, Angst DB (2006). Family patterns of decision-making in pediatric clinical trials. Res Nurs Health.

[20] Grigsby TJ, Kent EE, Montoya MJ, Sender LS, Morris RA, Ziogas A (2014). Attitudes toward cancer clinical trial participation in young adults with a history of cancer and a healthy college student sample: a preliminary investigation. J Adolesc Young Adult Oncol.

[21] Read K, Fernandez CV, Gao J, Strahlendorf C, Moghrabi A, Pentz RD (2009). Decision-making by adolescents and parents of children with cancer regarding health research participation. Pediatrics..

[22] Barakat LP, Schwartz LA, Reilly A, Deatrick JA, Balis F (2014). A qualitative study of phase III cancer clinical trial enrollment decision-making: perspectives from adolescents, young adults, caregivers, and providers. J Adolesc Young Adult Oncol.

[23] Bell JA, Forcina V, Mitchell L, Tam S, Wang K, Gupta AA (2018). Perceptions of and decision making about clinical trials in adolescent and young adults with Cancer: a qualitative analysis. BMC Cancer.

[24] Pearce S, Brownsdon A, Fern L, Gibson F, Whelan J, Lavender V (2018). The perceptions of teenagers, young adults and professionals in the participation of bone cancer clinical trials. Eur J Cancer Care.

[25] Ingersgaard MV, Tulstrup M, Schmiegelow K, Larsen HB (2018). A qualitative study of decision-making on phase III randomized clinical trial participation in paediatric oncology: adolescents’ and parents’ perspectives and preferences. J Adv Nurs.

[26] Tong A, Sainsbury S, Craig J (2007). Consolidated criteria for reporting qualitative research (COREQ): a 32-item checklist for interviews and focus groups. Int J Qual Health Care.

[27] Britten N (1995). Qualitative research: qualitative interviews in medical research. BMJ..

[28] Pope C, Mays N (1995). Qualitative research: reaching the parts other methods cannot reach: an introduction to qualitative methods in health and health services research. BMJ..

[29] Williams SJ (1999). Is anybody there? Critical realism, chronic illness and the disability debate. Sociol Health Illn.

[30] Lawton J (2003). Lay experiences of health and illness: past research and future agendas. Sociol Health Illn.

[31] Sandelowski M (2000). Whatever happened to qualitative description?. Res Nurs Health.

[32] Sandelowski M (2010). What’s in a name? Qualitative description revisited. Res Nurs Health.

[33] Rankin D, Harden J, Waugh N, Noyes K, Barnard KD, Stephen J (2014). Pathways to diagnosis: a qualitative study of the experiences and emotional reactions of parents of children diagnosed with type 1 diabetes. Pediatr Diabetes.

[34] Peel E, Parry O, Douglas M, Lawton J (2004). Diagnosis of type 2 diabetes: a qualitative analysis of patients’ emotional reactions and views about information provision. Patient Educ Couns.

[35] Strauss A, Corbin JM (1997). Grounded theory in practice.

[36] Alahmad G (2018). Informed consent in pediatric oncology: a systematic review of qualitative literature. Cancer Control.

[37] Buckley JM, Irving AD, Goodacre S (2016). How do patients feel about taking part in clinical trials in emergency care?. Emerg Med J.

[38] Woolfall Kerry, Young Bridget, Frith Lucy, Appleton Richard, Iyer Anand, Messahel Shrouk, Hickey Helen, Gamble Carrol (2014). Doing challenging research studies in a patient-centred way: a qualitative study to inform a randomised controlled trial in the paediatric emergency care setting. BMJ Open.

[39] Lawton J, Snowdon C, Morrow S, Norman JE, Denison FC, Hallowell N (2016). Recruiting and consenting into a peripartum trial in an emergency setting: a qualitative study of the experiences and views of women and healthcare professionals. Trials..

[40] Robertson EG, Wakefield CE, Signorelli C, Cohn RJ, Patenaude A, Foster C (2018). Strategies to facilitate shared decision-making about pediatric oncology clinical trial enrollment: a systematic review. Patient Educ Couns.

[41] Gillies K, Skea ZC, Campbell MK (2014). Decision aids for randomised controlled trials: a qualitative exploration of stakeholders’ views. BMJ Open.

[42] Politi MC, Kuzemchak MD, Kaphingst KA, Perkins H, Liu J, Byrne MM (2016). Decision aids can support cancer clinical trials decisions: results of a randomized trial. Oncologist.

[43] Baker JN, Leek AC, Salas HS, Drotar D, Noll R, Rheingold SR (2013). Suggestions from adolescents, young adults, and parents for improving informed consent in phase 1 pediatric oncology trials. Cancer..

[44] Hamilton DW, Heaven B, Thomson RG, Wilson JA, Exley C (2016). Multidisciplinary team decision-making in cancer and the absent patient: a qualitative study. BMJ Open.

[45] Dew K, Signal L, Stairmand J, Simpspon A, Sarfati D (2019). Cancer care decision-making and treatment consent: an observational study of patients’ and clinicians’ rights. J Sociol.

[46] Hahlweg P, Harter M, Nestoriuc Y, Scholl I (2017). How are decisions made in cancer care? A qualitative study using participant observation of current practice. BMJ Open.

[47] Ernst J, Berger S, Weißflog G, Schröder C, Körner A, Niederwieser D (2013). Patient participation in the medical decision-making process in haemato-oncology–a qualitative study. Eur J Cancer Care.

[48] Rosenbaum L (2015). The paternalism preference – choosing unshared decision making. N Engl J Med.

[49] Mol A (2008). The logic of care: health and the problem of patient choice.

[50] Charles C, Gafni A, Whelan T (1997). Shared decision-making in the medical encounter: what does it mean? (or it takes at least two to tango). Soc Sci Med.

[51] Edwards A, Elwyn G (2006). Inside the black box of shared decision making: distinguishing between the process of involvement and who makes the decision. Health Expect.

[52] Kars MC, Duijnstee MS, Pool A, Van Delden JJ, Grypdonck MH (2008). Being there: parenting the child with acute lymphoblastic leukaemia. J Clin Nurs.

[53] Holm KE, Patterson JM, Gurney JG (2003). Parental involvement and family-centered care in the diagnostic and treatment phases of childhood cancer: results from a qualitative study. J Pediatr Oncol Nurs.

