# Supplementary material for: The challenges of making informed decisions about treatment and trial participation following a cancer diagnosis: a qualitative study involving adolescents and young adults with cancer and their caregivers
Source: BMC Health Serv Res. 2020 Jan 8;20:25. doi: 10.1186/s12913-019-4851-1 (PMC6950988; doi:10.1186/s12913-019-4851-1)
Supplement: Supplementary file 1 — Additional file 1. Key areas explored in the AYA and caregiver interviews. [file 12913_2019_4851_MOESM1_ESM.docx]

**Additional File: Key areas explored in the AYA and caregiver interviews**

- Self, family and life before diagnosis
- Lead up to diagnosis
  - Changing health
  - Help-seeking
- When and how found out they (or son/daughter) had cancer
  - What told and by whom
  - Reaction(s) to diagnosis (AYA’s & caregivers’)
- Physical and emotional states
- Expectations of what was to come
  - Prior knowledge of cancer
- Initial conversations about treatment
  - Thoughts, questions, concerns (AYA’s & caregivers’)
- What needed to happen before treatment could begin
- Treatment / care-related decisions made, if any
  - What
  - When
  - How / By whom / Why
- Understanding of the term ‘clinical trial’
- If (AYA) invited to take part in a trial
  - When and by whom
  - Information given
  - Thoughts, questions, concerns (AYA’s & caregivers’)
  - Views on potential costs and benefits of participation
  - Decision
    - What
    - How made
    - Feelings about (AYA) being asked to decide
      - Sense of decisional capacity at that time
- If (AYA) not invited to take part in a trial
  - Anticipated feelings about (AYA) being approached soon after diagnosis
    - What might affect how felt
  - Probable thoughts, questions and concerns
  - Views on potential costs and benefits of participation
  - AYA’s likely decision
    - How would make such a decision (Would caregivers play a part?)
- Any unmet needs for information and support
- Advice for others
